# Short-type hydrophilic guidewire for reintervention after metal stents placement in malignant hilar biliary obstruction

**DOI:** 10.1055/a-2864-1329

**Published:** 2026-05-12

**Authors:** Hiroto Nishio, Naminatsu Takahara, Tatsuya Sato, Kensaku Noguchi, Kazunaga Ishigaki, Tomotaka Saito, Mitsuhiro Fujishiro

**Affiliations:** 1 Department of Gastroenterology in Organ Pathophysiology programGraduate School of Medicine, The University of TokyoTokyoJapan; 2 Department of Clinical Oncology 26782 The University of Tokyo HospitalTokyoJapan

The bilateral placement of self-expandable metal stents (SEMSs) is a widely accepted palliative treatment for unresectable malignant hilar biliary obstruction (MHBO), as adequate biliary drainage is associated with improved survival and prolonged stent patency 1 . Among the two major stenting methods, side-by-side and stent-in-stent (SIS), the SIS technique is often preferred because of its lower incidence of procedure-related adverse events, including pancreatitis 2 3 . However, endoscopic reintervention for recurrent biliary obstruction after multiple SIS SEMS placements remains technically challenging 4 .

Key steps in overcoming this challenge include selecting the SEMS mesh and inserting plastic stents (PSs) through the mesh with a guidewire (GW) support.

While stiff GWs provide a strong support for PS insertion, their limited flexibility can impair maneuverability within the mesh and increase the risk of the PS tip being trapped in the mesh, potentially causing insertion difficulty or SEMS damage ( Video 1 ). In contrast, hydrophilic GWs offer greater flexibility and mesh selectivity, reducing PS–SEMS interference and facilitating smoother stent insertion.

**Fig. 1:**
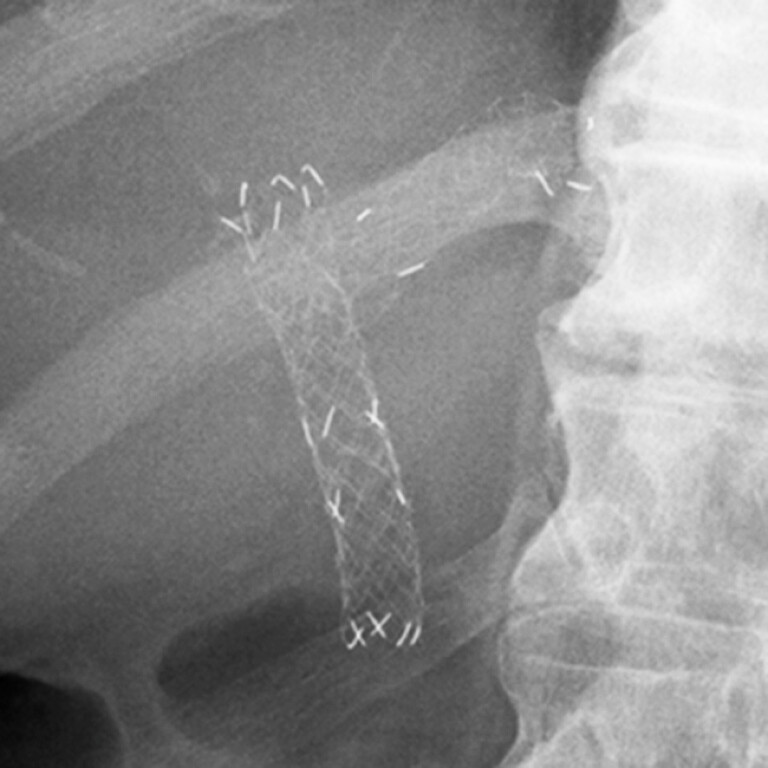
The previous placement of three SEMSs for malignant hilar biliary obstruction using the SIS technique. SEMS, self-expandable metal stent; SIS, stent-in-stent.

**Fig. 2:**
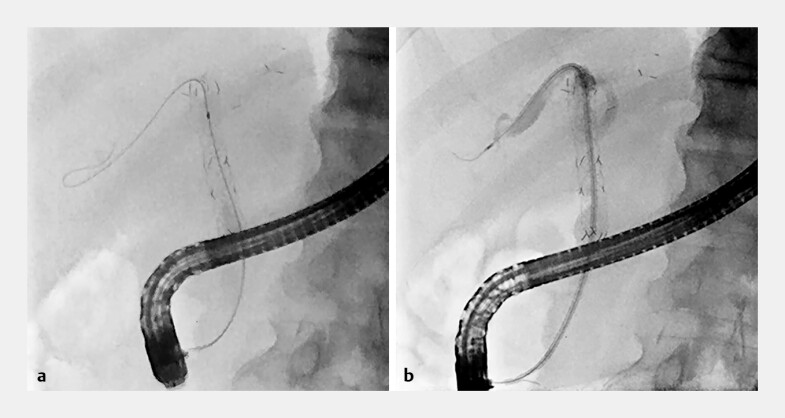
**a** Selecting an appropriate SEMS mesh using a short-type hydrophilic GW. **b** The PS was navigated smoothly without the PS–SEMS interference or SEMS damage. GW, guidewire; PS, plastic stent; SEMS, self-expandable metal stent.

**Fig. 3:**
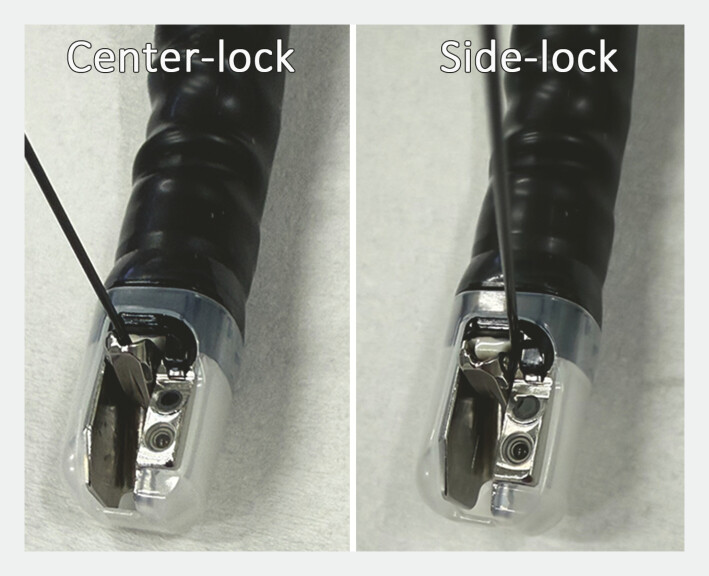
Comparison of center-lock and side-lock guidewire fixation mechanisms.

A 63-year-old man with unresectable hilar cholangiocarcinoma after the prior placement of three SEMSs using the SIS technique ( Fig. 1 ) was admitted for endoscopic reintervention. After selecting an appropriate SEMS mesh using a short-type hydrophilic GW (0.035-inch, 260-cm Radifocus; Terumo, Tokyo, Japan), gentle traction was applied to the GW to create a shaking motion, allowing the smooth insertion of a PS (Through & Pass Through the Mesh; Gadelius Medical, Tokyo, Japan) within the SEMS lumen ( Fig. 2 ). The “side-lock” technique, securing the GW alongside the elevator ( Fig. 3 ), facilitates stable device exchange, particularly when using a short-length GW. Finally, all PSs were inserted using a single GW ( Video 1 ).

Endoscopic reintervention using a short-type hydrophilic GW after the placement of SEMSs in malignant hilar biliary obstruction. GW, guidewire; SEMS, self-expandable metal stent.Video 1

A short-type hydrophilic GW combined with the “side-lock” technique enables safe and efficient reintervention after the SIS SEMS placement in MHBO.

Endoscopy_UCTN_Code_CCL_1AZ_2AC
